# Supplementary material for: A Comparative Phylogeography of Three Marine Species with Different PLD Modes Reveals Two Genetic Breaks across the Southern Caribbean Sea
Source: Animals (Basel). 2023 Aug 5;13(15):2528. doi: 10.3390/ani13152528 (PMC10417521; doi:10.3390/ani13152528)
Supplement: Supplementary file 1 [file animals-13-02528-s001.zip › animals-2399016-supplementary.pdf]

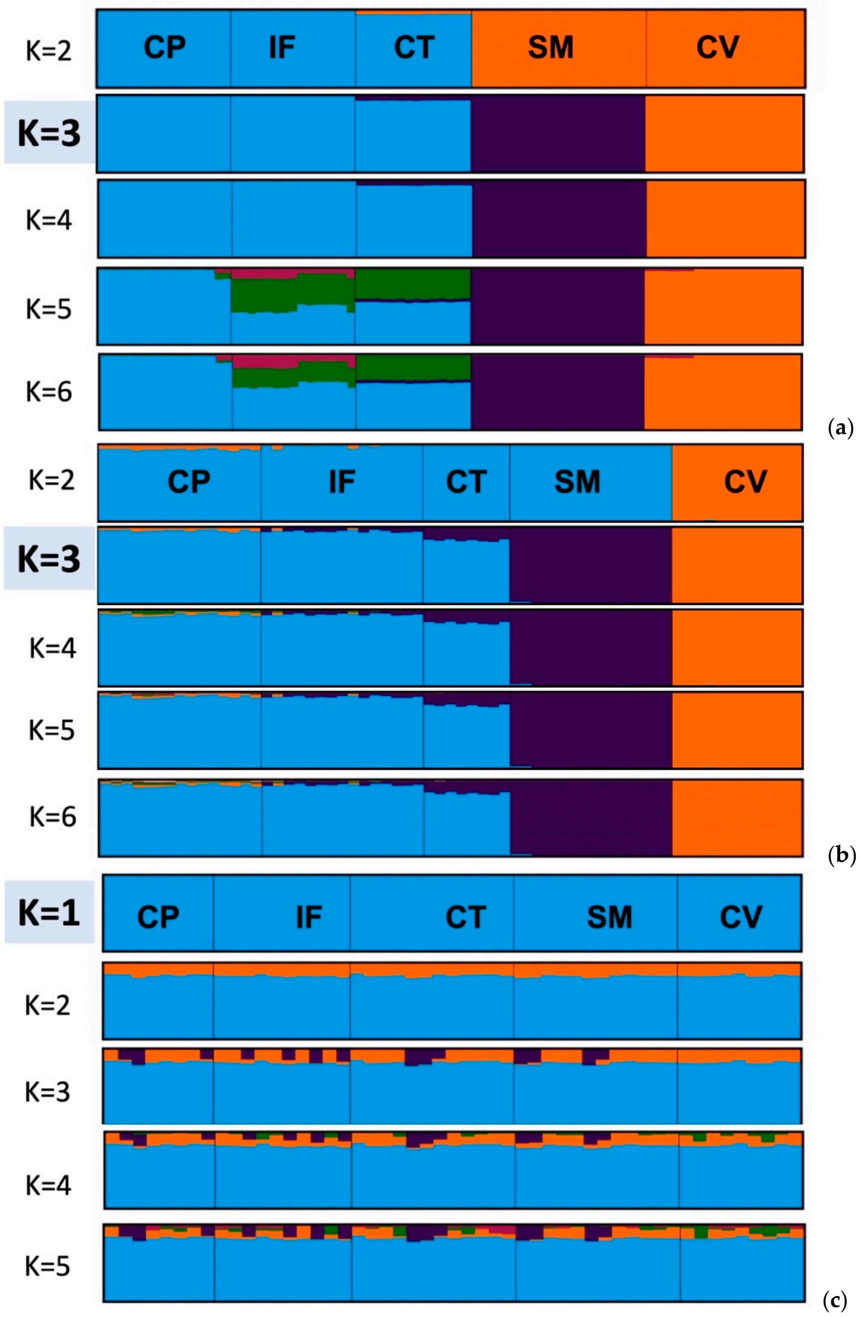

Figure S1. Bar graphs of the population ancestry coefficient from the STRUCTURE program indicating the probable number of populations (K = 2-6) for each species: (**a**) *Acanthemblemaria rivasi*, (**b**) *Cittarium pica*, and (**c**) *Nerita tessellata*.

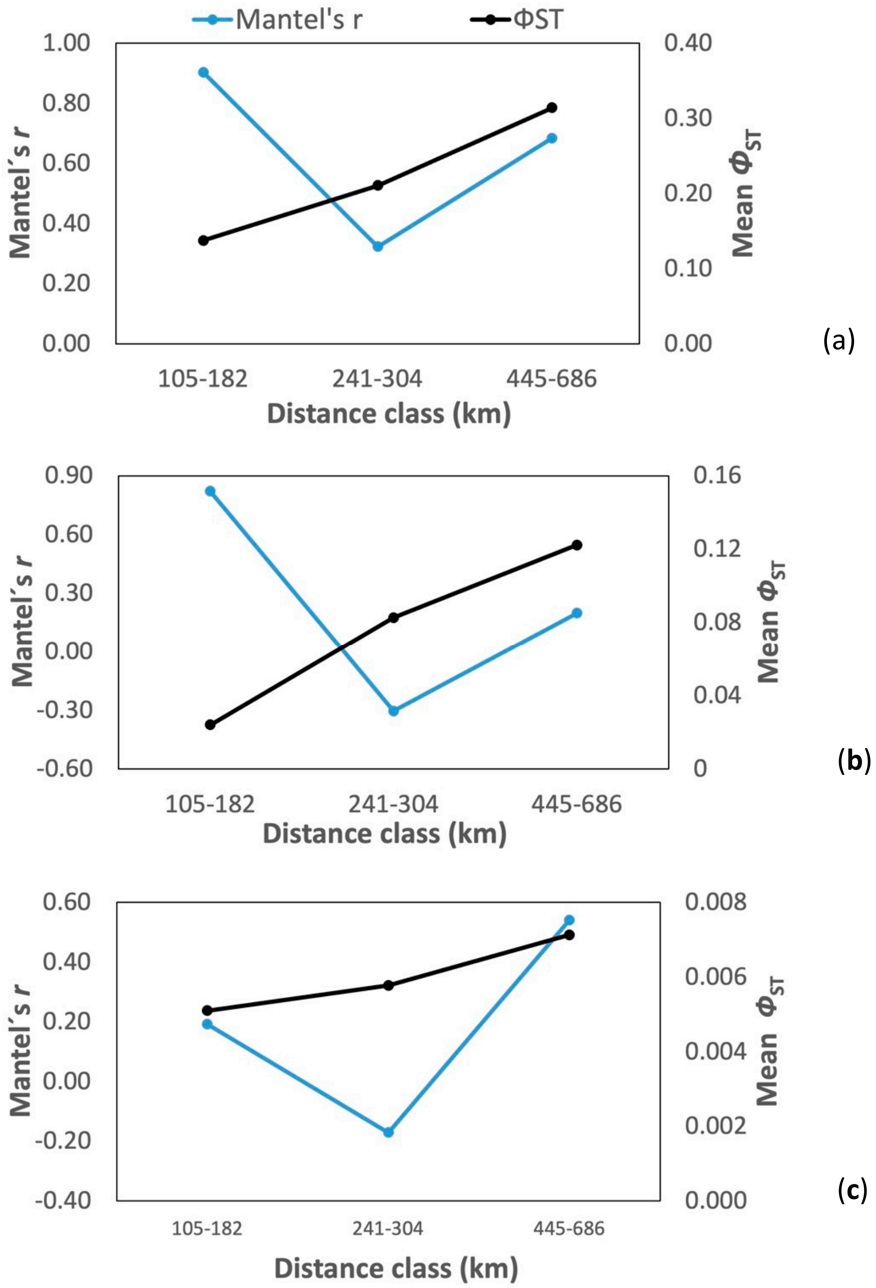

Figure S2. Correlogram plots of correlation coefficients and averages of estimated Φ_{ST} at each linear geographic distance class mark (right) for (**a**) *Acanthemblemaria rivasi*, (**b**) *Cittarium pica*, and (**c**) *Nerita tessellata*.

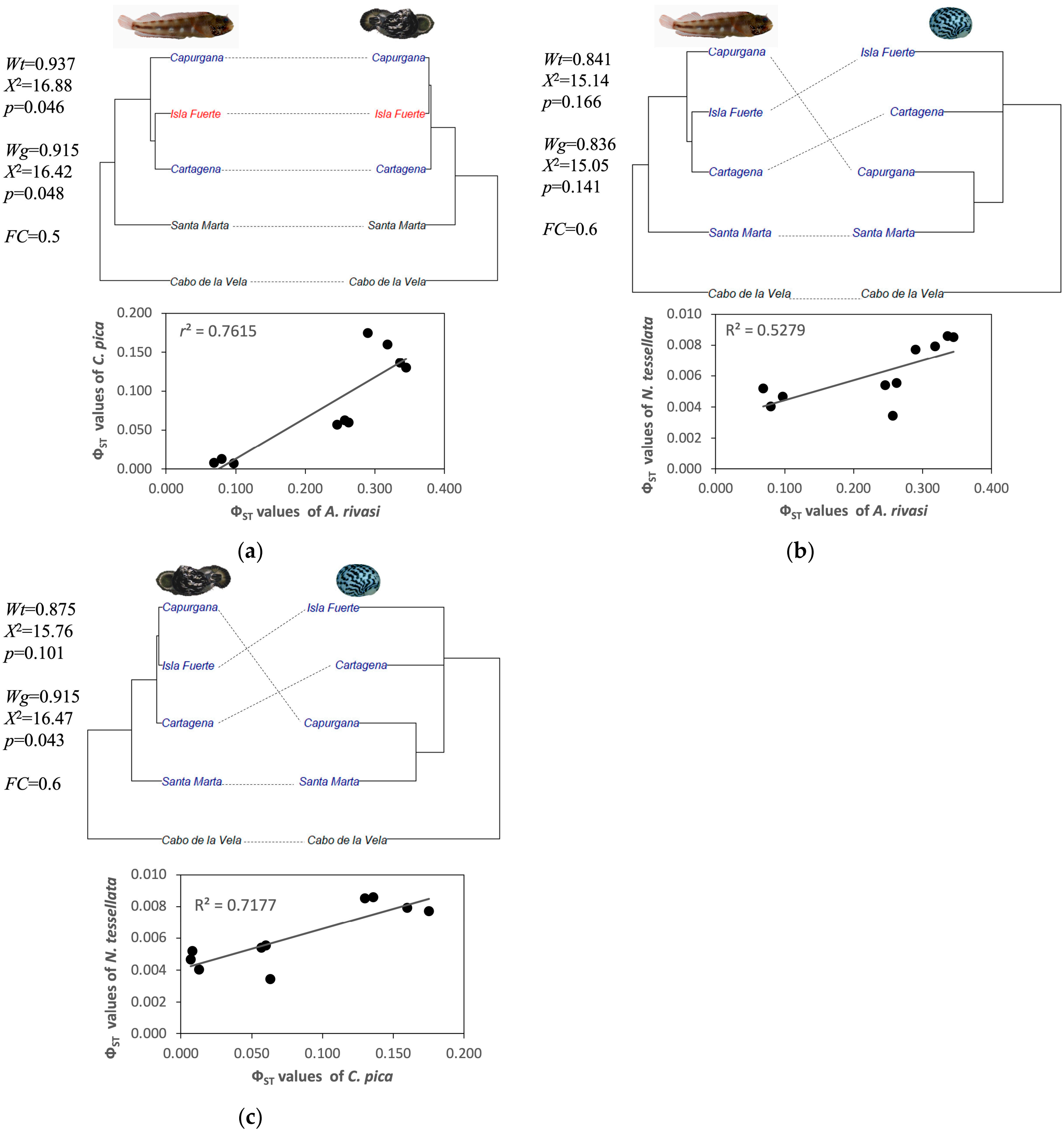

Figure S3. Dendrograms of Φ_{ST} values constructed with the UPGMA method for *A. rivasi*, *C. pica* and *N. tessellata*, and spatial autocorrelation analysis between the Φ_{ST} matrices relating the localities across the southern Caribbean, Colombia sector. (**a**) Analysis between *A. rivasi and C. pica*; (**b**) *A. rivasi* and *N. tessellata*; (**c**) *C. pica* and *N. tessellata*. *FC* = topological concordance factor; *W* = Kendall's statistic for topological (*Wt*) and genetic (*Wg*) congruence; *rm* = Mantel correlation coefficient; *p* = significance level. The black, blue, and red colors of the site names indicate high, medium, and low concordance among their locations in the dendrograms.

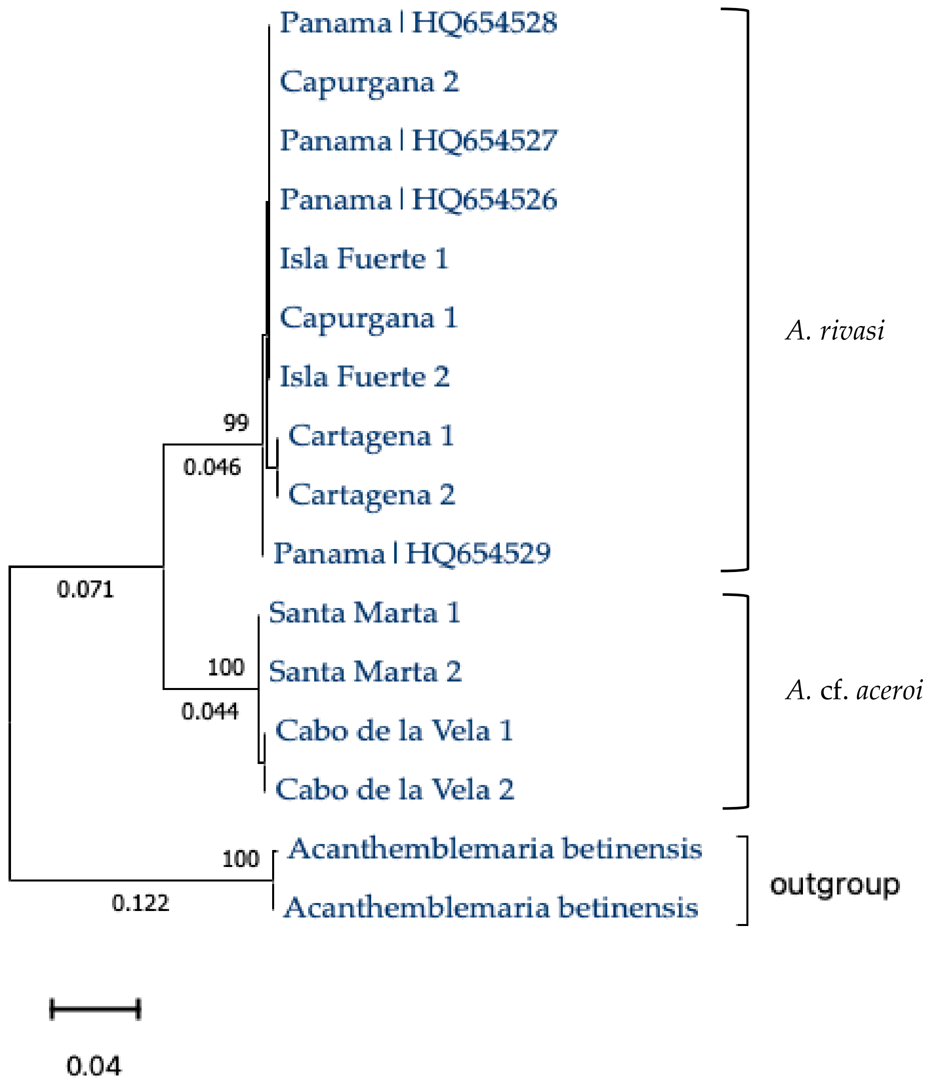

Figure S4. The phylogenetic analysis between COI gene sequences of samples from Colombian localities and Panama (*A. rivasi*). The analysis was inferred by using the Maximum Likelihood method and Hasegawa-Kishino-Yano model, with 1000 bootstrap replicates with the program MEGA XI. The length of branches and percentage of trees in which the associated samples clustered together is shown below and above the branches, respectively. The Panamanian samples were downloaded from GenBank with accession numbers: HQ654526.1, HQ654527.1, HQ654528.1, and HQ654529.1. The primers and PCR conditions for sequencing analysis were based on Ward et al. (2005).

Table S1. The genetic diversity indices calculated for *Acanthemblemaria rivasi*, *Cittarium pica*, and *Nerita tessellata* in five localities distributed across the southern Caribbean Sea, Colombia sector. Num: number of alleles, Eff_num: effective number of alleles, Ho: observed heterozygosity, Hs: heterozygosity within populations, and Gis: inbreeding coefficient. SD: standard deviation.

			Acanthemblemaria rivasi			
Localities	Number of samples	Num	Eff_num	Ho	Hs	Gis
Cabo de la Vela	19	1,222	1,097	0.063	0.062	-0.009
Santa Marta	21	1,404	1,206	0.121	0.129	0.069
Cartagena	14	1,537	1,211	0.132	0.141	0.066
Isla Fuerte	15	1,521	1,225	0.145	0.147	0.017
Capurganá	16	1,449	1,169	0.106	0.113	0.061
	Global value	2,000	1,243	0.163	0.181	0.045
	SD	0	0.001	0.001	0.001	0.001
			Cittarium pica			
Localities	Number of samples	Num	Eff_num	Ho	Hs	Gis
Cabo de la Vela	12	1,437	1,220	0.131	0.141	0.073
Santa Marta	15	1,629	1,282	0.165	0.182	0.097
Cartagena	8	1,632	1,283	0.170	0.193	0.120
Isla Fuerte	15	1,766	1,293	0.173	0.195	0.117
Capurganá	15	1,764	1,293	0.177	0.195	0.095
	Global value	2,000	1,243	0.163	0.181	0.102
	SD	0	0.001	0.001	0.001	0.001
			Nerita tessellata			
Localities	Number of samples	Num	Eff_num	Ho	Hs	Gis
Punta Gallinas	9	1,603	1,225	0.138	0.159	0.134
Santa Marta	12	1,702	1,232	0.141	0.162	0.130
Cartagena	12	1,666	1,226	0.139	0.159	0.127
Isla Fuerte	10	1,644	1,230	0.140	0.162	0.140
Capurganá	8	1,541	1,219	0.136	0.156	0.127
	Global value	2,000	1,213	0.139	0.16	0.131
	SD	0	0.002	0.001	0.001	0.002

Table S2. Estimates of evolutionary divergence between Panamanian COI sequences of *Acanthemblemaria rivasi* and Colombian localities, which were conducted using the Kimura 2-parameter model and 1000 bootstrap replicates with the program MEGA XI.

	A. cf. aceroi		***A. rivasi***					
**Localities/species**	**Cabo de la Vela**	**Santa Marta**	**Cartagena**	**Isla Fuerte**	**Capurganá**	**HQ654527**	**HQ654528**	**HQ654529**
Cabo de la Vela (*A. cf aceroi*)	0							
Santa Marta (*A. cf aceroi*)	0.003	0						
Cartagena (*A. rivasi*)	0.102	0.098	0					
Isla Fuerte (*A. rivasi*)	0.096	0.092	0.005	0				
Capurganá (*A. rivasi*)	0.096	0.092	0.005	0.000	0			
HQ654527 (*A. rivasi*)	0.096	0.092	0.005	0.000	0.000	0		
HQ654528 (*A. rivasi*)	0.097	0.093	0.005	0.000	0.000	0.000	0	
HQ654529 (*A. rivasi*)	0.094	0.090	0.007	0.002	0.002	0.002	0.002	0
